# The Role of Th17 Response in COVID-19

**DOI:** 10.3390/cells10061550

**Published:** 2021-06-19

**Authors:** Diana Martonik, Anna Parfieniuk-Kowerda, Magdalena Rogalska, Robert Flisiak

**Affiliations:** Department of Infectious Diseases and Hepatology, Medical University of Bialystok, 15-540 Bialystok, Poland; anna.parfieniuk@umb.edu.pl (A.P.-K.); magda.rogalska@umb.edu.pl (M.R.); robert.flisiak1@gmail.com (R.F.)

**Keywords:** COVID-19 pneumonia, SARS-CoV-2, cytokines, Th17 response

## Abstract

COVID-19 is an acute infectious disease of the respiratory system caused by infection with the SARS-CoV-2 virus (Severe Acute Respiratory Syndrome Coronavirus 2). Transmission of SARS-CoV-2 infections occurs through droplets and contaminated objects. A rapid and well-coordinated immune system response is the first line of defense in a viral infection. However, a disturbed and over-activated immune response may be counterproductive, causing damage to the body. Severely ill patients hospitalised with COVID-19 exhibit increased levels of many cytokines, including Interleukin (IL)-1β, IL-2, IL-6, IL-7, IL-8, IL-10, IL-17, granulocyte colony stimulating factor (G-CSF), monocyte chemoattractant protein 1 (MCP-1) and tumor necrosis factor (TNF). Increasing evidence suggests that Th17 cells play an important role in the pathogenesis of COVID-19, not only by activating cytokine cascade but also by inducing Th2 responses, inhibiting Th1 differentiation and suppressing Treg cells. This review focuses on a Th17 pathway in the course of the immune response in COVID-19, and explores plausible targets for therapeutic intervention.

## 1. Introduction

COVID-19 is an acute infectious disease of the respiratory system caused by infection with the SARS-CoV-2 virus (Severe Acute Respiratory Syndrome Coronavirus 2), belonging to the coronavirus family. Coronaviruses (CoVs) are a large group of enveloped, positive-strand RNA viruses that infect mammals and birds. So far, seven species of human pathogenic CoVs (HCoVs) have been identified. Two of these, with high virulence Severe Acute Respiratory Syndrome Coronavirus (SARS-CoV) and Middle East Respiratory Syndrome-related Coronavirus (MERS-CoV), caused widespread epidemics, which were limited by sanitary and epidemiological control methods. Other HCoVs are a common (15–30%) cause of upper respiratory tract infections in humans [1]. Transmission of SARS-CoV-2 infections occurs through droplets and contaminated objects. Due to the presence of the virus in many bodily fluids (including nasal and pharyngeal secretions, sputum, tears and blood), other transmission routes are not excluded [2]. The incubation period is five days, and the symptoms include fever, cough, dyspnea and muscle pain [3].

Upon hospital admission, patients exhibit many abnormalities in laboratory test results, including a decreased lymphocyte, monocyte and platelet count, as well as an increased level of C-reactive protein (CRP), D-dimers, lactate dehydrogenase (LDH), alanine transaminase (ALT) and aspartate transaminase (AST) [4]. Chest CT findings in patients with COVID-19 pneumonia include ground glass opacities (GGOs), crazy paving patterns and peripheral consolidation [5,6].

Cytokines play an important role in the immunopathology of viral infections. A rapid and well-coordinated immune system response is the first line of defense in a viral infection. However, a disturbed and over-activated immune response may be counterproductive, causing damage to the body. Excessive activation of immune system in response to both infectious and non-infectious agents causing production of pro-inflammatory cytokines is described as “cytokine storm syndrome” (CSS), an acute inflammatory reaction that leads to multiple organ failure. Severely ill patients hospitalised with COVID-19 exhibit increased levels of Interleukin (IL)-1β, IL-2, IL-6, IL-7, IL-8, IL-10, IL-17, granulocyte colony stimulating factor (G-CSF), monocyte chemoattractant protein 1 (MCP-1) and tumor necrosis factor (TNF) [1,3,7,8,9,10,11,12,13].

So far, CSS recognition criteria have not been well established, and the term is used for critically ill patients with high levels of pro-inflammatory cytokines [3]. Increasing evidence suggests that Th17 cells play an important role in the pathogenesis of COVID-19, not only by activating cytokine cascade but also by inducing Th2 responses, inhibiting Th1 differentiation and suppressing Treg cells [14,15].

The present review focuses on a Th17 pathway in the course of the immune response in COVID-19, and explores plausible targets for therapeutic intervention.

## 2. Mechanisms of SARS-CoV-2 Invasion

There are four distinguishable structural proteins: the spike (S), the membrane (M), the nucleocapsid (N) and the envelope (E) protein in SARS-CoV-2 structure. Furthermore, in SARS-CoV-2 infection, the entry-point receptor on the host cell is also angiotensin-converting enzyme 2 (ACE2). Interaction of SARS-CoV-2 spike protein via the receptor-bind domains (RBDs) with the cell receptor ACE2 induces the endocytosis, and enables entry into many different types of cells, including type II alveolar epithelial cells, monocytes and macrophages. Priming and activation of S protein by the transmembrane serine protease (TMPRSS) 2, TMPRSS4, Factor Xa or by cathepsin is needed in order to facilitate cell surface entry, thus enabling the fusion of SARS-CoV-2 virus with the host cells and viral replication [16,17]. It has been shown that SARS-CoV-2 has 10–20 times higher binding affinity to ACE2 than SARS-CoV, which may result in more effective viral transmission through droplets from individuals with COVID-19. More efficacious transmission and a longer incubation period may explain a much greater number of cases infected than of Severe Acute Respiratory Syndrome (SARS) [7,18,19].

The role of ACE2 is the conversion of angiotensin II (Ang II) into angiotensin_(1–7)_ (Ang_(1–7)_), which shows cardiovascular protective function through its vasodilatory, anti-inflammatory and antiproliferative effects. SARS-CoV-2 infection causes a decrease in ACE2 expression, resulting in the inhibition of its regulatory and protective functions. Moreover, the downregulation of ACE2 causes an increase of Ang II concentrations in the serum, which in addition to being a vasoconstrictor, has proliferative and pro-inflammatory properties [7,20,21,22,23].

The ACE2 is expressed in most tissues, but the highest expression has been found in the endothelial cells of blood vessels and epithelial cells of the respiratory tract, gastrointestinal tract and kidneys. Therefore, there is a variety of cell lines susceptible to SARS-CoV-2 [7,20,21]. New evidence suggests that among these, the highest replication rate occurs in pulmonary and intestinal cell lines. Furthermore, significant replication occurs in the hepatic and renal cells lines [18].

## 3. T Cells Differentiation

Naïve CD4^+^ T cells differentiate into several subgroups of effector cells, depending on their function and released cytokines. The main subsets are T helper type 1 (Th1), T helper type 2 (Th2), T helper type 17 (Th17) and regulatory T (Treg) cells [24].

Increased expression of signal transducer and activator of transcription 1 (STAT1) and T-box transcription factor 21 (TBX21) induce Th1 phenotype cells and production of interleukin 2 (IL-2), interferon-γ (IFN-γ) and tumor necrosis factor (TNF). Th1 cells are responsible for the mediation of immune responses against intracellular pathogens by promoting the activation of macrophages, B cells, NK cells and CD8^+^ T cells. Even though Th1 cells are essential for the clearance of intracellular pathogens, exaggerated Th1 response have been associated with diseases such as rheumatoid arthritis and multiple sclerosis [25,26]. Recent studies in COVID-19 provided evidence for the beneficial role of Th1 responses directed against SARS-CoV-2 for the resolution of symptoms and infection in convalescent individuals. In contrast, Th2 cells activated by the increased expression of STAT6 and GATA binding protein 3 (GATA3) produce IL-4, IL-5, IL-10 and IL-13, and are responsible for the mediation of immune responses against extracellular pathogens by promoting the activation of eosinophils, basophils and mast cells. Dysregulation of Th2 responses have been associated with the exacerbation of allergic reactions and autoimmune diseases such as systemic lupus erythematosus [25,27]. Th17 cells, induced by RAR-related orphan receptor (ROR) γt, RORα and STAT3, produce IL-17A, IL-17F, IL-21 and IL-22, and have been suggested to be crucial for autoimmune inflammation [24]. In comparison to the Th1 immune responses, the antigen-specific Th2 or Th17 responses were not detected in a convalescent state, since they have been suggested to play a role in the immune-driven lung injury and contributed to the ARDS progression through facilitating neutrophil recruitment [28,29].

In contrast, Treg cells are essential for inhibiting immune responses by suppressing the activity of a variety of cells. Tregs are induced by forkhead box protein 3 (Foxp3), and produce anti-inflammatory cytokines TGF-β and IL-10 [30,31].

The direction of differentiation depends on many factors, but the most important role is played by the cytokines present during activation. Cytokines promoting Th1 cell differentiation are mainly IL-12 and IFN-γ, while IL-4 and IL-2 contribute to Th2 cell formation [24]. Many cytokines contribute to the differentiation of Th17 cells. Naïve CD4^+^ cells differentiate into Th17 under the synergistic exposure to TGF-β and IL-6. In addition, IL-21 and IL-23 contribute to the formation of pathogenic Th17 cells, while IFN-γ, IL-2 and IL-4 inhibit the process [32].

## 4. Th17 Lineage

In the immune system, Th17 cells have both a protective and pathogenic role. Therefore, the Th17 phenotype has been associated with chronic inflammation and autoimmune diseases. A wide array of cytokines, including IL-17A, IL-17F, IL-21, IL-22, granulocyte-macrophage colony-stimulating factor (GM-CSF), IL-10 and IFN-γ are produced by the Th17 subset [30,32,33].

IL-17 attracts neutrophils and monocytes to the infected tissue and induces production of cytokines like G-CSF and IL-6 that promote innate inflammation and chemokines such as the C-X-C motif chemokine ligand (CXCL) 1, CXCL2 and CXCL10, which recruit myeloid cells to the site of infection. IL-17 is produced mainly by CD4^+^, CD8^+^ and innate lymphoid cells (ILCs), but under certain circumstances may be also produced by neutrophils. Studies have shown increased levels of IL-17A in patients with diseases such as psoriasis, asthma, rheumatoid arthritis and multiple sclerosis [34,35,36].

IL-21 plays an important role in the differentiation and function of T follicular helper (Tfh) and Th17 cells. Furthermore, IL-21 is responsible for differentiation of B cells into plasma cells and the enhancement of immunoglobulin production. IL-21 has been associated with diseases such as systemic lupus erythematosus, primary Sjogren’s syndrome, type 1 diabetes and psoriasis [37,38,39,40].

In contrast, IL-22 promotes proliferation in non-hematopoietic epithelial cells and fibroblasts in many tissues, including skin, lungs and the gastrointestinal tract. In addition, IL-22 is involved in tissue regeneration and the regulation of defense mechanisms at barrier surfaces. Nonetheless, increased concentrations of IL-22 have been demonstrated in patients with ulcerative colitis and Crohn’s disease. Moreover, IL-22 is strongly linked to cancer in many sites, including the skin, lung and colon, by its contribution to tumor growth and malignancy [41,42,43].

GM-CSF is produced at inflammation sites, and is involved in the proliferation of myeloid cells from progenitor cells. Furthermore, evidence shows its importance in managing functions of mature myeloid cells. Increased levels of GM-CSF have been found in patients with lung and colorectal cancer, rheumatoid arthritis and multiple sclerosis [44,45,46,47].

IFN-γ plays a crucial role in the clearance of virally infected cells through the promotion of cytotoxic T-cell responses. Additionally, IFN-γ is responsible for the activation of macrophages to produce a wide array of inflammatory mediators and increase tumoricidal activity of NK cells. Nevertheless, IFN-γ has been associated with autoimmune diseases such as dermatomyositis, rheumatoid arthritis and systemic lupus erythematosus [48,49,50,51].

## 5. IL-23 Influence on Th17 Pathogenicity

**Figure 1:**
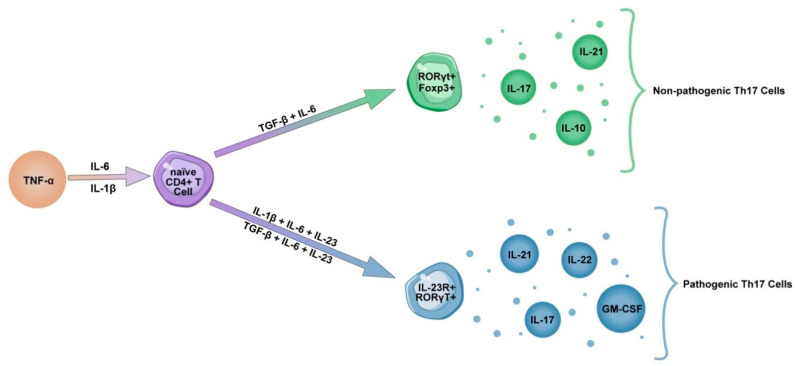
Development of pathogenic Th17 cells. Synergistic exposure to TGF-β and IL-6 induces transcription factors RORγt and Foxp3 to produce IL-17, IL-21 and an anti-inflammatory cytokine IL-10. Another way of inducing Th17 cells is synergistic exposure to IL-6 and IL-1β, which are produced under influence of TNFα. However, exposure to IL-23 induces receptor IL-23R to produce IL-17, IL-21, IL-22 and GM-CSF, therefore making Th17 cells pathogenic. Furthermore, it has been shown that IL-23 reduces the concentrations of IL-10, additionally contributing to Th17 pathogenicity.

As previously mentioned, the synergistic influence of TGF-β and IL-6, inducing transcription factor RORγt, is needed for the development of Th17 cells. However, it has been demonstrated that exposure to IL-23 is essential for expansion of Th17 cells, and triggers their pathogenicity (Figure 1). IL-23 is a heterodimeric pro-inflammatory cytokine formed by a unique subunit p19, and shared with IL-12, subunit p40. IL-23 is produced by monocytes, macrophages and dendritic cells, as well as signals through transcriptional factors IL12-Rβ1 and IL-23R. In pathological states, excessive IL-23 signaling induces the production of pathogenic mediators, such as IL-17, IL-22, GM-CSF and TNFα promoting the recruitment of monocytes and granulocytes causing damage at the inflammation site, thus inducing chronic inflammation and the development of clinical symptoms [52,53,54,55,56,57].

## 6. Th17 and Tc17 Cells in COVID-19

Increasing evidence suggests that the Th17 inflammatory response plays an important role in the pathogenesis of COVID-19 pneumonia. Exacerbation of the immune response occurs through the release of cytokines such as IL-17 and GM-CSF, the promotion of neutrophil migration and the downregulation of the Treg response. Unlike Th17 cells, Treg cells express anti-inflammatory mediators (IL-4, IL-10 and TGF-β) and play an important role in weakening overactive immune responses [35]. The Treg/Th17 cell ratio is decreased in patients with severe COVID-19 due to the decreased number of Treg cells, indicating the insufficient regulation of pro-inflammatory responses [58,59]. Furthermore, prior evidence shows that the Treg/Th17 balance is associated with the severity of uncontrolled systemic inflammation in Acute Lung Injury (ALI) and Acute Respiratory Distress Syndrome (ARDS) [60,61]. Therefore, the dysregulation of the Treg/Th17 cells ratio skewing towards the Th17 phenotype may contribute to the uncontrolled release of cytokine and chemokine cascades in COVID-19 patients, leading to aggravated inflammatory responses and tissue damage [35]. Several studies demonstrated increased levels of IL-17 and GM-CSF in peripheral blood and tears of patients with COVID-19, and a higher fraction of Th17 cells in bronchoalveolar lavage fluid of these patients [11,62,63,64,65,66,67,68,69]. Similarly, robust Th17 responses were observed in patients with MERS-CoV and SARS-CoV infections [70,71,72]. A strong Th17 response was also observed in H1N1 influenza virus infection [73]; moreover, prior evidence associated IL-17 with Acute Respiratory Distress Syndrome and Neonatal Respiratory Distress Syndrome (NRDS) [74,75]. Increased concentrations of IL-17 were found in plasma and alveolar fluid of patients with ARDS. In addition, when compared to survivors, significantly higher levels of IL-17 were found in a group of non-survivors. Furthermore, a negative correlation between the PaO_2_/FiO_2_ ratio and level of IL-17 was found in these patients [76]. Interestingly, it has been shown that in macaques infected with the simian immunodeficiency virus, the percentage of CD161^+^CD8^+^ Tc17 cells producing IL-17 in lung tissue was four times higher than in peripheral blood. Additionally, these cells could secrete more IL-17 than those present in peripheral blood [77]. Thus, IL-17 might promote pulmonary inflammation, following the infection by neutrophil and monocyte migration to the lungs, and by activating other cytokine cascades (G-CSF, TNFα, IL-1β and IL-6) [11,78,79]. In addition, plasma from COVID-19 patients revealed a fourfold increase of the IFN-γ levels, which activates macrophages to produce proinflammatory cytokines, indicating a Th1/Th17 response [11,80,81]. Elevated levels of IFN-γ were also found in MERS-CoV infections [82]. Moreover, prior evidence shows that high concentrations of IFN-γ in rapidly progressive interstitial lung disease associated with dermatomyositis positively correlated with the ground-glass opacity score (G-score) in CT [51].

Studies have shown increased levels of IL-21 and IL-22 in plasma of patients hospitalised with COVID-19 [83,84]. Moreover, prior evidence shows that IL-22 plays a crucial role in LPS-induced ALI [85]. Interestingly, higher concentrations of IL-21 and IFN-γ have been also demonstrated in COVID-19 convalescent plasma [86].

Taken together, the evidence supports the involvement of a Th17 mediated response in the pathogenesis of pneumonia caused by SARS-CoV-2. Therefore, targeting the Th17 phenotype might be beneficial in patients with a dominant Th17 response.

## 7. Possible Treatment Strategy

Currently, there are several biological drugs targeting IL-17 (secukinab, brodalumab, ixekizumab) and IL-23 (guselkumab, gildrakizumab and risankizumab) approved for the treatment of rheumatologic and dermatologic diseases such as psoriasis. Furthermore, there are biological drugs that intervene in the cell differentiation towards Th17 phenotype through blocking STAT3, which seems to be crucial for the production of IL-17A cytokine. STAT3 activation is mediated by IL-6 and IL-23 through Janus kinase (JAK) 2 signaling pathway, and by IL-21 through the JAK1 and JAK3 signaling pathways. Since IL-23 triggers the pathogenicity of Th17 cells, blocking JAK signaling pathways may restrict the expansion of pathogenic Th17 cells [62]. Biologics can increase the risk of infection. However, there have been reports of psoriatic patients receiving biologic treatment with mild or asymptomatic COVID-19 manifestation, suggesting that IL-17 and IL-23 inhibitors may play a protective role in the setting of SARS-CoV-2 infection [87,88,89]. Decreased injury, such as pulmonary edema and leukocytes infiltration into alveoli, was demonstrated in the murine model of LPS-induced Acute Lung Injury after IL-17 antibody administration. Moreover, ALI mice showed decreased concentrations of TNFα and increased concentrations of anti-inflammatory IL-10 in plasma and alveolar fluid after the administration of the IL-17 blocking agent [76]. Evidence shows that the delivery of Th17 inhibiting therapy might prove effective in preventing lung tissue destruction. Therefore, more research is needed to assess the practicality of using this type of therapy in patients with COVID-19. There is currently a randomised phase II clinical trial to evaluate the efficacy and safety of secukinab, in addition to standard treatment for the management of novel coronavirus pneumonia registered in the Brazilian Clinical Trial Registry (RBR-5vpyh4). Another multicenter clinical trial investigating the use of ixekizumab in combination with anti-viral therapy in patients with severe COVID-19 infection has been registered in the Chinese Clinical Trial Registry (ChiCTR2000030703). In addition, there is a phase III randomised clinical trial to evaluate the efficacy and safety of ixekizumab in combination with aldesleukin in patients with severe to critical COVID-19 (NCT04724629). Moreover, there are several clinical trials to evaluate JAK inhibitors. In the phase III multicenter clinical trial held in North and South America and Europe, ruxolitinib, a selective inhibitor of JAK1/JAK2, failed to reduce the number of COVID-19-hospitalised patients who experienced severe complications (RUXCOVID, NCT04362137). The German study on ruxolitinib in patients with COVID-19-induced ARDS (RuXoCoil, NCT04359290) is still under assessment. Another JAK inhibitor, baricitinib, has shown beneficial effects for COVID-19 treatment in combination with remdesivir in a global multicenter clinical study (NCT04401579). However, more research is needed to confirm its usefulness for the treatment of severe COVID-19 pneumonia.

## 8. Conclusions

Several pro-inflammatory mediators may promote the exacerbation of immune response causing hyperinflammation, and the current challenge is understanding how they modulate specific pathological situations to find suitable treatment. Patients with severe COVID-19 exhibit high levels of IL-17 and GM-CSF. Moreover, it has been shown that lung tissue destruction might be explained by recruitment of neutrophils mediated by Th17 cells. Thus, targeting Th17 response in the early stages of COVID-19 may alleviate the course of the disease and improve clinical outcomes.

## Data Availability

Not applicable.

## Abbreviations

ACE2	Angiotensin-converting enzyme 2
ALI	Acute Lung Injury
ALT	Alanine transaminase
ARDS	Acute Respiratory Distress Syndrome
AST	Aspartate transaminase
CRP	C-reactive protein
CSS	Cytokine Storm Syndrome
CXCL	The C-X-C motif chemokine ligand
GATA3	GATA binding protein 3
G-CSF	Granulocyte colony stimulating factor
GGO	Ground-glass opacity
GM-CSF	Granulocyte-macrophage colony-stimulating factor
Foxp3	Forkhead box protein 3
IL	Interleukin
ILC	Innate lymphoid cell
JAK	Janus kinase
LDH	Lactate dehydrogenase
MCP-1	Monocyte chemoattractant protein 1
MERS-CoV	Middle East Respiratory Syndrome-related Coronavirus
NRDS	Neonatal Respiratory Distress Syndrome
RBD	Receptor-bind domain
ROR	RAR-related orphan receptor
SARS	Severe Acute Respiratory Syndrome
SARS-CoV	Severe Acute Respiratory Syndrome Coronavirus
STAT	Signal transducer and activator of transcription
Tbet	T-box transcription factor TBX21
TNF	Tumor necrosis factor

## References

[1] Catanzaro M., Fagiani F., Racchi M., Corsini E., Govoni S., Lanni C. (2020). Immune response in COVID-19: Addressing a pharmacological challenge by targeting pathways triggered by SARS-CoV-2. Signal Transduct. Target. Ther..

[2] Chan J.F.W., Yuan S., Kok K.H., To K.K.W., Chu H., Yang J., Xing F., Liu J., Yip C.C.Y., Poon R.W.S. (2020). A familial cluster of pneumonia associated with the 2019 novel coronavirus indicating person-to-person transmission: A study of a family cluster. Lancet.

[3] Tang Y., Liu J., Zhang D., Xu Z., Ji J., Wen C. (2020). Cytokine Storm in COVID-19: The Current Evidence and Treatment Strategies. Front. Immunol..

[4] Shen B., Yi X., Sun Y., Bi X., Du J., Zhang C., Quan S., Zhang F., Sun R., Qian L. (2020). Proteomic and Metabolomic Characterization of COVID-19 Patient Sera. Cell.

[5] Akçay Ş., Özlü T., Yilmaz A. (2020). Radiological approaches to COVID-19 pneumonia. Turk. J. Med. Sci..

[6] Hu L., Wang C. (2020). Radiological role in the detection, diagnosis and monitoring for the coronavirus disease 2019 (COVID-19). Eur. Rev. Med. Pharmacol. Sci..

[7] Hojyo S., Uchida M., Tanaka K., Hasebe R., Tanaka Y., Murakami M., Hirano T. (2020). How COVID-19 induces cytokine storm with high mortality. Inflamm. Regen..

[8] Bordoni V., Sacchi A., Cimini E., Notari S., Grassi G., Tartaglia E., Casetti R., Giancola M.L., Bevilacqua N., Maeurer M. (2020). An Inflammatory Profile Correlates with Decreased Frequency of Cytotoxic Cells in Coronavirus Disease 2019. Clin. Infect. Dis..

[9] Li H., Liu L., Zhang D., Xu J., Dai H., Tang N., Su X., Cao B. (2020). SARS-CoV-2 and viral sepsis: Observations and hypotheses. Lancet.

[10] Tay M.Z., Poh C.M., Rénia L., Macary P.A., Ng L.F.P. (2020). The trinity of COVID-19: Immunity, inflammation and intervention. Nat. Rev. Immunol..

[11] De Biasi S., Meschiari M., Gibellini L., Bellinazzi C., Borella R., Fidanza L., Gozzi L., Iannone A., Tartaro D.L., Mattioli M. (2020). Marked T cell activation, senescence, exhaustion and skewing towards TH17 in patients with COVID-19 pneumonia. Nat. Commun..

[12] Schultheiß C., Paschold L., Simnica D., Mohme M., Willscher E., von Wenserski L., Scholz R., Wieters I., Dahlke C., Tolosa E. (2020). Next-Generation Sequencing of T and B Cell Receptor Repertoires from COVID-19 Patients Showed Signatures Associated with Severity of Disease. Immunity.

[13] Ye Q., Wang B., Mao J. (2020). The pathogenesis and treatment of the ‘Cytokine Storm’ in COVID-19. J. Infect..

[14] Toh M.-L., Kawashima M., Zrioual S., Hot A., Miossec P., Miossec P. (2009). IL-17 inhibits human Th1 differentiation through IL-12Rβ2 downregulation. Cytokine.

[15] Wang Y.-H., Liu Y.-J. (2008). The IL-17 cytokine family and their role in allergic inflammation. Curr. Opin. Immunol..

[16] Costa L.B., Perez L.G., Palmeira V.A., E Cordeiro T.M., Ribeiro V.T., Lanza K., E Silva A.C.S. (2020). Insights on SARS-CoV-2 Molecular Interactions with the Renin-Angiotensin System. Front. Cell Dev. Biol..

[17] Kreutzberger A.J.B., Sanyal A., Ojha R., Pyle J.D., Vapalahti O., Balistreri G., Kirchhausen T. (2021). Synergistic inhibition of two host factors that facilitate entry of Severe Acute Respiratory Syndrome Coronavirus 2. bioRxiv.

[18] Chu H., Chan J.F.-W., Yuen T.T.-T., Shuai H., Yuan S., Wang Y., Hu B., Yip C.C.-Y., Tsang J.O.-L., Huang X. (2020). Comparative tropism, replication kinetics, and cell damage profiling of SARS-CoV-2 and SARS-CoV with implications for clinical manifestations, transmissibility, and laboratory studies of COVID-19: An observational study. Lancet Microbe.

[19] Wrapp D., Wang N., Corbett K.S., Goldsmith J.A., Hsieh C.-L., Abiona O., Graham B.S., McLellan J.S. (2020). Cryo-EM structure of the 2019-nCoV spike in the prefusion conformation. Science.

[20] Anguiano L., Riera M., Pascual J., Soler M.J. (2017). Circulating ACE2 in Cardiovascular and Kidney Diseases. Curr. Med. Chem..

[21] Touyz R.M., Montezano A.C. (2018). Angiotensin-(1–7) and Vascular Function. Hypertension.

[22] Benigni A., Cassis P., Remuzzi G. (2010). Angiotensin II revisited: New roles in inflammation, immunology and aging. EMBO Mol. Med..

[23] Chung M.K., Karnik S., Saef J., Bergmann C., Barnard J., Lederman M.M., Tilton J., Cheng F., Harding C.V., Young J.B. (2020). SARS-CoV-2 and ACE2: The biology and clinical data settling the ARB and ACEI controversy. EBioMedicine.

[24] Murphy K.M., Ouyang W., Farrar J.D., Yang J., Ranganath S., Asnagli H., Afkarian M., Murphy T.L. (2000). Signaling and Transcription in T Helper Development. Annu. Rev. Immunol..

[25] Luckheeram R.V., Zhou R., Verma A.D., Xia B. (2012). CD4^+^T Cells: Differentiation and Functions. Clin. Dev. Immunol..

[26] Dardalhon V., Korn T., Kuchroo V.K., Anderson A.C. (2008). Role of Th1 and Th17 cells in organ-specific autoimmunity. J. Autoimmun..

[27] Paul W.E., Zhu J. (2010). How are TH2-type immune responses initiated and amplified?. Nat. Rev. Immunol..

[28] Neidleman J., Luo X., Frouard J., Xie G., Gill G., Stein E.S., McGregor M., Ma T., George A.F., Kosters A. (2020). SARS-CoV-2-Specific T Cells Exhibit Phenotypic Features of Helper Function, Lack of Terminal Differentiation, and High Proliferation Potential. Cell Rep. Med..

[29] Hotez P.J., Bottazzi M.E., Corry D.B. (2020). The potential role of Th17 immune responses in coronavirus immunopathology and vaccine-induced immune enhancement. Microbes Infect..

[30] Lee G.R. (2018). The balance of Th17 versus treg cells in autoimmunity. Int. J. Mol. Sci..

[31] Ali N., Rosenblum M.D. (2017). Regulatory T cells in skin. Immunology.

[32] Bedoya S.K., Lam B., Lau K., Larkin J. (2013). Th17 Cells in Immunity and Autoimmunity. Clin. Dev. Immunol..

[33] Gaffen S.L., Jain R., Garg A.V., Cua D.J. (2014). The IL-23–IL-17 immune axis: From mechanisms to therapeutic testing. Nat. Rev. Immunol..

[34] Gu C., Wu L., Li X. (2013). IL-17 family: Cytokines, receptors and signaling. Cytokine.

[35] Muyayalo K.P., Huang D., Zhao S., Xie T., Mor G., Liao A. (2020). COVID-19 and Treg/Th17 imbalance: Potential relationship to pregnancy outcomes. Am. J. Reprod. Immunol..

[36] McGeachy M.J., Cua D.J., Gaffen S.L. (2019). The IL-17 Family of Cytokines in Health and Disease. Immunity.

[37] Liu S.M., King C. (2013). IL-21–Producing Th Cells in Immunity and Autoimmunity. J. Immunol..

[38] Spolski R., Leonard W.J. (2014). Interleukin-21: A double-edged sword with therapeutic potential. Nat. Rev. Drug Discov..

[39] Long D., Chen Y., Wu H., Zhao M., Lu Q. (2019). Clinical significance and immunobiology of IL-21 in autoimmunity. J. Autoimmun..

[40] Tian Y., Zajac A.J. (2016). IL-21 and T Cell Differentiation: Consider the Context. Trends Immunol..

[41] Dudakov J.A., Hanash A.M., Brink M.R.V.D. (2015). Interleukin-22: Immunobiology and Pathology. Annu. Rev. Immunol..

[42] Parks O.B., Pociask D.A., Hodzic Z., Kolls J.K., Good M. (2016). Interleukin-22 Signaling in the Regulation of Intestinal Health and Disease. Front. Cell Dev. Biol..

[43] Eyerich K., DiMartino V., Cavani A. (2017). IL-17 and IL-22 in immunity: Driving protection and pathology. Eur. J. Immunol..

[44] Shiomi A., Usui T., Mimori T. (2016). GM-CSF as a therapeutic target in autoimmune diseases. Inflamm. Regen..

[45] Becher B., Tugues S., Greter M. (2016). GM-CSF: From Growth Factor to Central Mediator of Tissue Inflammation. Immunity.

[46] Aliper A.M., Frieden-Korovkina V.P., Buzdin A., Roumiantsev S., Zhavoronkov A. (2014). A role for G-CSF and GM-CSF in nonmyeloid cancers. Cancer Med..

[47] Avci A.B., Feist E., Burmester G.-R. (2016). Targeting GM-CSF in rheumatoid arthritis. Clin. Exp. Rheumatol..

[48] Tau G., Rothman P.B. (1999). Biologic functions of the IFN-gamma receptors. Allergy.

[49] Ivashkiv L.B. (2018). IFNγ: Signalling, epigenetics and roles in immunity, metabolism, disease and cancer immunotherapy. Nat. Rev. Immunol..

[50] Kak G., Raza M., Tiwari B.K. (2018). Interferon-gamma (IFN-γ): Exploring its implications in infectious diseases. Biomol. Concepts.

[51] Ishikawa Y., Iwata S., Hanami K., Nawata A., Zhang M., Yamagata K., Hirata S., Sakata K., Todoroki Y., Nakano K. (2018). Relevance of interferon-gamma in pathogenesis of life-threatening rapidly progressive interstitial lung disease in patients with dermatomyositis. Arthritis Res..

[52] Bettelli E., Korn T., Oukka M., Kuchroo V.K. (2008). Induction and effector functions of TH17 cells. Nat. Cell Biol..

[53] McGeachy M.J., Bak-Jensen K.S., Chen Y., Tato C.M., Blumenschein W.M., McClanahan T.K., Cua D.J. (2007). TGF-β and IL-6 drive the production of IL-17 and IL-10 by T cells and restrain TH-17 cell–mediated pathology. Nat. Immunol..

[54] Lee Y., Awasthi A., Yosef N., Quintana F.J., Xiao S., Peters A., Wu C., Kleinewietfeld M., Kunder S., Hafler D.A. (2012). Induction and molecular signature of pathogenic TH17 cells. Nat. Immunol..

[55] Bettelli E., Korn T., Kuchroo V.K. (2007). Th17: The third member of the effector T cell trilogy. Curr. Opin. Immunol..

[56] Tsukazaki H., Kaito T. (2020). The Role of the IL-23/IL-17 Pathway in the Pathogenesis of Spondyloarthritis. Int. J. Mol. Sci..

[57] Pastor-Fernández G., Mariblanca I.R., Navarro M.N. (2020). Decoding IL-23 Signaling Cascade for New Therapeutic Opportunities. Cells.

[58] Qin C., Zhou L., Hu Z., Zhang S., Yang S., Tao Y., Xie C., Ma K., Shang K., Wang W. (2020). Dysregulation of Immune Response in Patients with Coronavirus 2019 (COVID-19) in Wuhan, China. Clin. Infect. Dis..

[59] Neurath M.F. (2020). COVID-19 and immunomodulation in IBD. Gut.

[60] Lin S., Wu H., Wang C., Xiao Z., Xu F. (2018). Regulatory T Cells and Acute Lung Injury: Cytokines, Uncontrolled Inflammation, and Therapeutic Implications. Front. Immunol..

[61] Yu Z.-X., Ji M.-S., Yan J., Cai Y., Liu J., Yang H.-F., Li Y., Jin Z.-C., Zheng J.-X. (2015). The ratio of Th17/Treg cells as a risk indicator in early acute respiratory distress syndrome. Crit. Care.

[62] Wu D., Yang X.O. (2020). TH17 responses in cytokine storm of COVID-19: An emerging target of JAK2 inhibitor Fedratinib. J. Microbiol. Immunol. Infect..

[63] Burgos-Blasco B., Güemes-Villahoz N., Santiago J.L., Fernandez-Vigo J.I., Espino-Paisán L., Sarriá B., García-Feijoo J., Martinez-De-La-Casa J.M. (2020). Hypercytokinemia in COVID-19: Tear cytokine profile in hospitalized COVID-19 patients. Exp. Eye Res..

[64] Petrone L., Petruccioli E., Vanini V., Cuzzi G., Fard S.N., Alonzi T., Castilletti C., Palmieri F., Gualano G., Vittozzi P. (2021). A whole blood test to measure SARS-CoV-2-specific response in COVID-19 patients. Clin. Microbiol. Infect..

[65] Ghazavi A., Ganji A., Keshavarzian N., Rabiemajd S., Mosayebi G. (2021). Cytokine profile and disease severity in patients with COVID-19. Cytokine.

[66] Weiskopf D., Schmitz K.S., Raadsen M.P., Grifoni A., Okba N.M.A., Endeman H., Akker J.P.C.V.D., Molenkamp R., Koopmans M.P.G., Van Gorp E.C.M. (2020). Phenotype and kinetics of SARS-CoV-2-specific T cells in COVID-19 patients with acute respiratory distress syndrome. Sci. Immunol..

[67] Xu Z., Shi L., Wang Y., Zhang J., Huang L., Zhang C., Liu S., Zhao P., Liu H., Zhu L. (2020). Pathological findings of COVID-19 associated with acute respiratory distress syndrome. Lancet Respir. Med..

[68] Pasrija R., Naime M. (2021). The deregulated immune reaction and cytokines release storm (CRS) in COVID-19 disease. Int. Immunopharmacol..

[69] Ronit A., Berg R.M., Bay J.T., Haugaard A.K., Ahlström M.G., Burgdorf K.S., Ullum H., Rørvig S.B., Tjelle K., Foss N.B. (2021). Compartmental immunophenotyping in COVID-19 ARDS: A case series. J. Allergy Clin. Immunol..

[70] Mahallawi W.H., Khabour O.F., Zhang Q., Makhdoum H.M., Suliman B.A. (2018). MERS-CoV infection in humans is associated with a pro-inflammatory Th1 and Th17 cytokine profile. Cytokine.

[71] Faure E., Poissy J., Goffard A., Fournier C., Kipnis E., Titecat M., Bortolotti P., Martinez L., Dubucquoi S., Dessein R. (2014). Distinct Immune Response in Two MERS-CoV-Infected Patients: Can We Go from Bench to Bedside?. PLoS ONE.

[72] Josset L., Menachery V.D., Gralinski L.E., Agnihothram S., Sova P., Carter V.S., Yount B.L., Graham R.L., Baric R.S., Katze M.G. (2013). Cell Host Response to Infection with Novel Human Coronavirus EMC Predicts Potential Antivirals and Important Differences with SARS Coronavirus. mBio.

[73] Bermejo-Martin J.F., De Lejarazu R.O., Pumarola T., Rello J., Almansa R., Ramírez P., Martin-Loeches I., Varillas D., Gallegos M.C., Serón C. (2009). Th1 and Th17 hypercytokinemia as early host response signature in severe pandemic influenza. Crit. Care.

[74] Mikacenic C., Hansen E.E., Radella F., Gharib S.A., Stapleton R.D., Wurfel M.M. (2016). Interleukin-17A Is Associated With Alveolar Inflammation and Poor Outcomes in Acute Respiratory Distress Syndrome. Crit. Care Med..

[75] Zheng L.-Y., Sun P.-C. (2020). Increased Expression of IL-23 and IL-17 in Serum of Patients with Neonatal Respiratory Distress Syndrome and its Clinical Significance. Clin. Lab..

[76] Ding Q., Liu G.-Q., Zeng Y.-Y., Zhu J.-J., Liu Z.-Y., Zhang X., Huang J.-A. (2017). Role of IL-17 in LPS-induced acute lung injury: An in vivo study. Oncotarget.

[77] Rout N. (2016). Enhanced Th1/Th17 Functions of CD161^+^ CD8^+^ T Cells in Mucosal Tissues of Rhesus Macaques. PLoS ONE.

[78] McCarthy M.K., Zhu L., Procario M.C., Weinberg J.B. (2014). IL-17 contributes to neutrophil recruitment but not to control of viral replication during acute mouse adenovirus type 1 respiratory infection. Virology.

[79] Lindén A., Laan M., Anderson G.P. (2005). Neutrophils, interleukin-17A and lung disease. Eur. Respir. J..

[80] Liu J., Li S., Liu J., Liang B., Wang X., Wang H., Li W., Tong Q., Yi J., Zhao L. (2020). Longitudinal characteristics of lymphocyte responses and cytokine profiles in the peripheral blood of SARS-CoV-2 infected patients. EBioMedicine.

[81] Han H., Ma Q., Li C., Liu R., Zhao L., Wang W., Zhang P., Liu X., Gao G., Liu F. (2020). Profiling serum cytokines in COVID-19 patients reveals IL-6 and IL-10 are disease severity predictors. Emerg. Microbes Infect..

[82] Yao Z., Zheng Z., Wu K., Junhua Z. (2020). Immune environment modulation in pneumonia patients caused by coronavirus: SARS-CoV, MERS-CoV and SARS-CoV-2. Aging.

[83] Croci S., Bonacini M., Dolci G., Massari M., Facciolongo N., Pignatti E., Pisciotta A., Carnevale G., Negro A., Cassone G. (2021). Human Dental Pulp Stem Cells Modulate Cytokine Production in vitro by Peripheral Blood Mononuclear Cells from Coronavirus Disease 2019 Patients. Front. Cell Dev. Biol..

[84] Lucas C., Team Y.I., Wong P., Klein J., Castro T.B.R., Silva J., Sundaram M., Ellingson M.K., Mao T., Oh J.E. (2020). Longitudinal analyses reveal immunological misfiring in severe COVID-19. Nat. Cell Biol..

[85] Sakaguchi R., Chikuma S., Shichita T., Morita R., Sekiya T., Ouyang W., Ueda T., Seki H., Morisaki H., Yoshimura A. (2015). Innate-like function of memory Th17 cells for enhancing endotoxin-induced acute lung inflammation through IL-22. Int. Immunol..

[86] Bonny T.S., Patel E.U., Zhu X., Bloch E.M., Grabowski M.K., Abraham A.G., Littlefield K., Shrestha R., E Benner S., Laeyendecker O. (2021). Cytokine and Chemokine Levels in Coronavirus Disease 2019 Convalescent Plasma. Open Forum Infect. Dis..

[87] Conti A., Lasagni C., Bigi L., Pellacani G. (2020). Evolution of COVID-19 infection in four psoriatic patients treated with biological drugs. J. Eur. Acad. Dermatol. Venereol..

[88] Messina F., Piaserico S. (2020). SARS-CoV-2 infection in a psoriatic patient treated with IL-23 inhibitor. J. Eur. Acad. Dermatol. Venereol..

[89] Balestri R., Rech G., Girardelli C. (2020). SARS-CoV-2 infection in a psoriatic patient treated with IL-17 inhibitor. J. Eur. Acad. Dermatol. Venereol..

